# Supplementary material for: Green Extraction at Scale: Hydrodynamic Cavitation for Bioactive Recovery and Protein Functionalization—A Narrative Review
Source: Molecules. 2026 Jan 5;31(1):192. doi: 10.3390/molecules31010192 (PMC12787678; doi:10.3390/molecules31010192)
Supplement: Supplementary file 1 [file molecules-31-00192-s001.zip › molecules-4011376-revised-Supplementary.pdf]

molecules

Review

Green Extraction at Scale: Hydrodynamic Cavitation for Bioactives Recovery and Protein Functionalization. A Narrative Review

Francesco Meneguzzo [1,*], Federica Zabini [1,†] and Lorenzo Albanese [1,†]

[1] Institute of Bioeconomy, National Research Council of Italy, Via Madonna del Piano 10, 50019 Firenze, Italy; federica.zabini@cnr.it (F.Z.); lorenzo.albanese@cnr.it (L.A.)
* Correspondence: francesco.meneguzzo@cnr.it; Tel.: +39-392-9850002
† These authors contributed equally to this work

Supplementary Materials

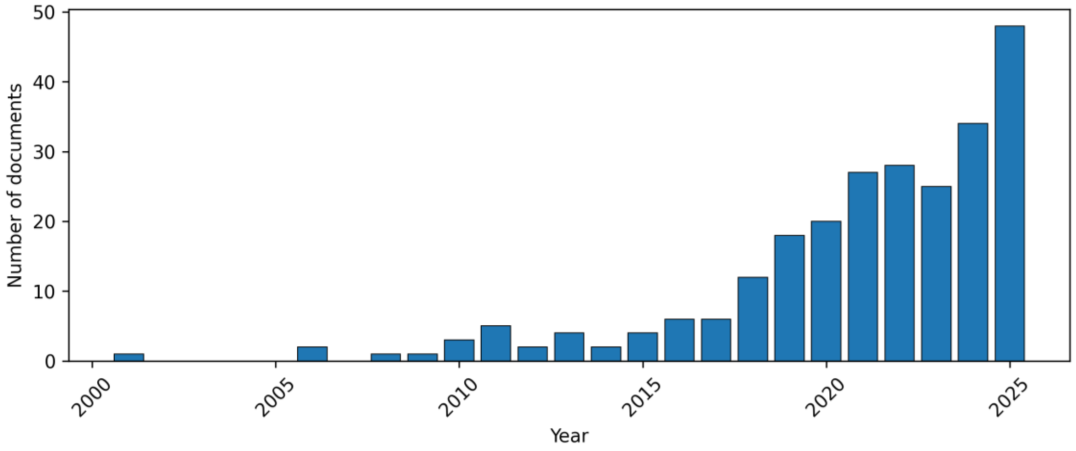

Figure S1. Number of Scopus documents per year returned by the query (2000–2025; n=249).

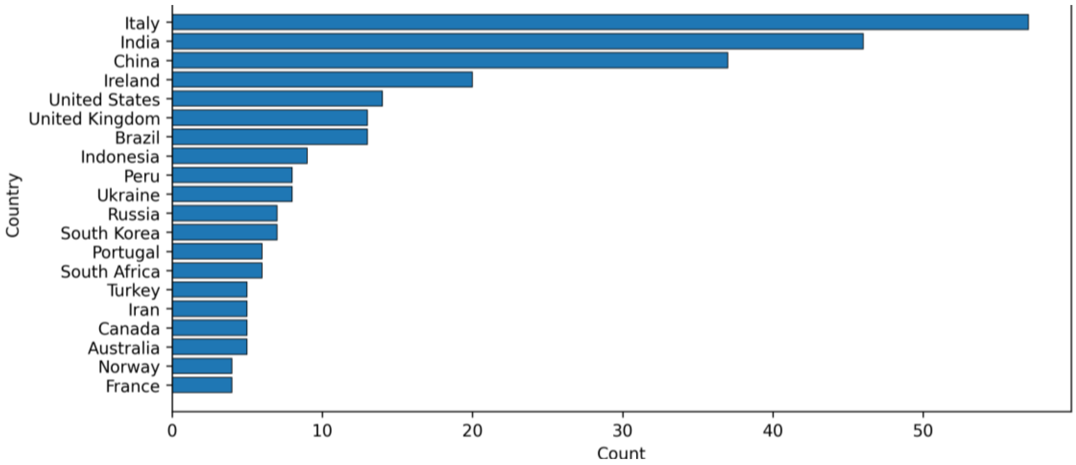

Figure S2. Most frequent countries in affiliations (top 20), parsed from the Scopus Affiliations field (paper-level counts; a paper may contribute to multiple countries).

Table S2. Main parameters used for the calculation of COGS for red orange peel dry extract.

Parameter	Level	Unit
Effective dose (daily amount)	200	mg
Raw biomass moisture	75	%
Water-to-biomass (fresh basis) ratio	2:1	
Extraction yield	30	%
Peak processing temperature	45	°C
Annual amount of dry extract	200	kg
Amount of carriers in spray-drying	30	%
Depreciation time of technological system	7	years
Number of shared productions [a]	3	

[a] The same processing system is used for different production lines (e.g., red orange peel, pomegranate peel, *Abies* alba byproducts).

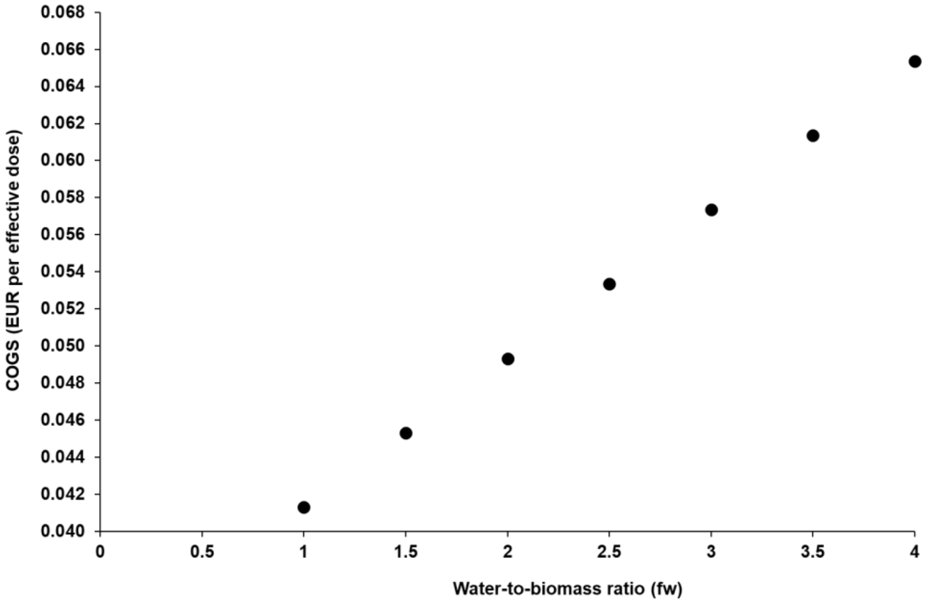

Figure S3. COGS per effective dose (illustrative model) as a function of the water-to-biomass ratio (fw).

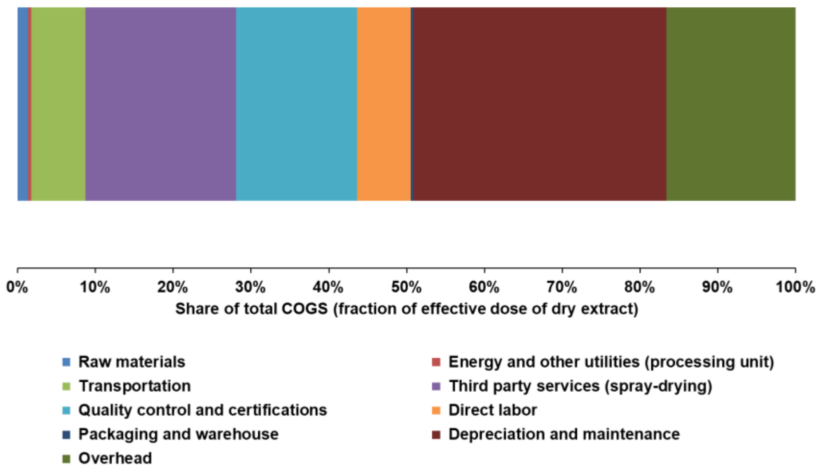

(**a**)

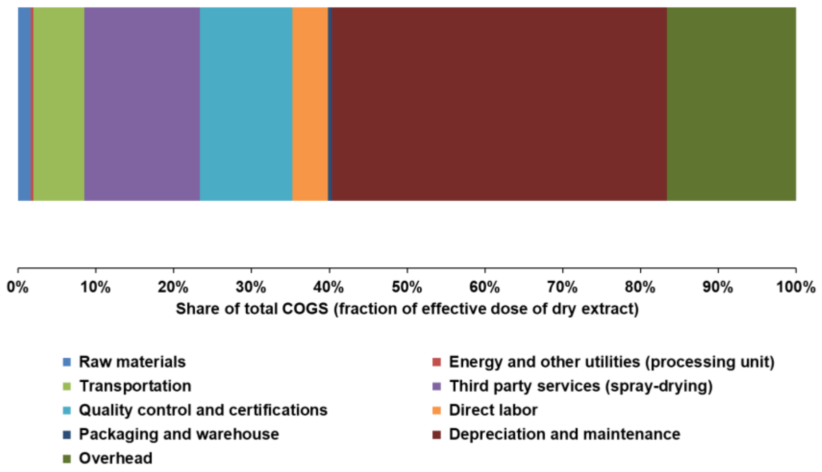

(**b**)

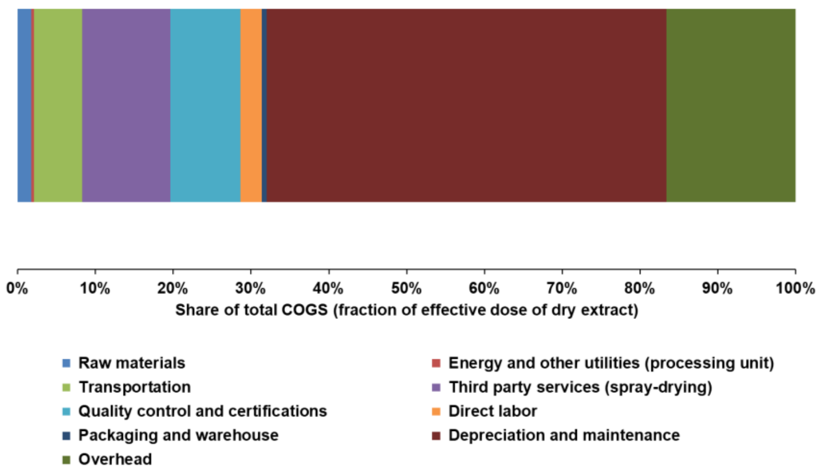

(**c**)

Figure S4. Category-level COGS contribution per effective dose (illustrative model): (**a**) water-to-biomass ratio (fw) = 4, with COGS = EUR 0.065; (**b**) water-to-biomass ratio (fw) = 2, with COGS = EUR 0.049; (**c**) water-to-biomass ratio (fw) = 1, with COGS = EUR 0.041.
